# Chemopreventive effects of *Strobilanthes crispus* leaf extract on azoxymethane-induced aberrant crypt foci in rat colon

**DOI:** 10.1038/srep13312

**Published:** 2015-08-26

**Authors:** Nawal Al-Henhena, Shaden A. M. Khalifa, Rozaida Poh Yuen Ying, Pouya Hassandarvish, Elham Rouhollahi, Nahla Saeed Al-Wajeeh, Habibah Mohd Ali, Mahmood Ameen Abdulla, Hesham R. El-Seedi

**Affiliations:** 1 Department of Biomedical Science, Faculty of Medicine, University of Malaya, 50603 Kuala Lumpur , Malaysia; 2 Department of Experimental Hematology, Karolinska University Hospital, SE-141 86 Stockholm, Sweden; 3 Department of Chemistry, Faculty of Science, University of Malaya, 50603 Kuala Lumpur, Malaysia; 4 Division of Pharmacognosy, Department of Medicinal Chemistry, Uppsala University, Box 574, SE-75 123, Uppsala, Sweden

## Abstract

In this work, microscopic and histological studies suggest that *Strobilanthes crispus* ethanol extract reduce azoxymethane (AOM)-induced colonic aberrant crypt foci (ACF) in rats. *S*. c*rispus* is considered a traditional medicine and used as an antioxidant. Its leaf contains a large amount of phenolic compounds to which its radical scavenging role is attributed and enhance its ability to eradicate oxidative stress reactions. The study was designed to determine the chemopreventive effect of *S. crispus* ethanol extract *in vivo* and *in vitro* by elucidating the effect of the extract on intermediate biomarkers which can be used as effective predictors of colon cancer. *S. crispus* was analyzed for DPPH free radical scavenging, nitric oxide (NO) and ferric acid reduction. The results indicated that *S. crispus* oral administration significantly inhibited colorectal carcinogenesis induced by AOM as revealed by the reduction in the number of ACF. *S. crispus* down-regulated the expression of PCNA, Bcl2 and β-catenin. Additionally, it exerted a pronounced inhibitory effect on MDA and NO levels and stimulatory effect on CAT and GPx activities. These results demonstrate that *S. crispus* is a chemopreventive agent for colorectal cancer through the suppression of early and intermediate carcinogenic phases that may be related to its flavonoid content.

Colorectal cancer is quite common and undoubtedly a major health problem among the Asian population^1^. The development of colorectal cancer is directly attributed to the activation of oxidative stress pathways in the intestinal lumen, which is manifested by the increased number and size of aberrant crypt foci. Aberrant crypt foci are benign adenomatous polyps that develop into an advanced adenoma with high-grade dysplasia and then progress to an invasive cancer^2^. The abundant appearance of aberrant crypt foci (ACF) as a precursor lesion and their increase in number are considered early abnormalities that occur during colorectal cancer induction^3^. Elevation of biochemical markers and consequent pathological signs are considered late markers associated with the imbalance between cellular growth and death and, hence, the progressive alteration of the intestinal homeostasis.

Oxidative stress plays a crucial role in the molecular mechanism of cancer development and progression^4^. Many studies have shown that oxidative radicals are among the contributing factors involved in cancer formation, invasion and metastatic spread. Exposure to oxidants may lead to enhanced expression of the enzyme nitric oxide synthase, resulting in an increase in the cellular metabolic rate. The oxidative radicals react with protein tyrosine and kinase residues and cause a second wave of lipid peroxidation. Lipid peroxidation activates a programmed death cascade and has been identified as a source of erroneous DNA replication, leading to gene mutation, mitochondrial membrane leakage, cell cycle transition and apoptosis (programmed cell death)^5^.

People are increasingly becoming aware that healthy nutrients cleanse their bodies from free radicals and improve their well-being. Nutrients with calcium, folate, fiber, omega-3 fatty acids, vitamin D, and vitamin A are highly appreciated by practitioners and healers as chemopreventive and chemotherapeutic mediators^6^,^7^. Dietary phytochemicals are proving to be a relatively simple and practical approach to a healthy life, and the daily consumption of flavonoid-rich vegetables or fruits has been recommended to reduce cancer risk. The wide range of chemical components that are naturally present in these dietary products has been associated with oxidative radical scavenging effects^8^,^9^. Flavonoid-rich phytochemicals from green tea are emerging as therapeutic agents for cancer cases in preclinical and clinical studies^10^. Their pharmacological effects relevant to cancer control have also been reported in the *American Journal of Clinical Nutrition* in which Lorenz 2013^11^ highlighted the active constituents of plant ethanol extracts and pinpointed the relevant mechanism of action. This research has been conducted in the context of chemotherapeutic treatments^12^, and the ethanol extracts have not consistently been shown to reduce the mortality rate of colorectal cancer cases, although tumor size control has been suggested. For instance, the five-year survival rate is only 10% in patients with metastatic colorectal cancer, even with the heavy use of the most common chemotherapy, fluoropyrimidine 5-fluorouracil (5-FU). Nevertheless, fluoropyrimidine 5-fluorouracil (5-FU) has some side effects and has shown cytotoxicity to normal cells^13^, as it irreversibly inhibits thymidylate synthase (TS).

In the present study, we wanted to extend the potential benefits of diets based on flavonoids for cancer prevention. In particular, we evaluated the chemopreventive effects of *Strobilanthes crispus*, with an emphasis on an azoxymethane (AOM)-induced ACF model. *S. crispus* (Family of *Acanthaceae*) is native to countries such as Malaysia, Madagascar and Indonesia. *S. crispus* is commonly known as “pecah beling” or “jin batu” in the Malay language. The consumption of nutraceutical *S. crispus* herbal tea is increasing due to the additional nutrients and antioxidants it provides. The recommended daily level of consumption of the leafy ethanol extract is 5 g. *S. crispus* herbal tea is known for its diuretic, laxative and antidiabetic properties^14^. It contains highly bioactive compounds^15^, which are reported to be responsible for its antioxidant^16^ and antibacterial effects^17^ and wound healing^18^; its anticancer^19^ and specifically anti-breast and anti-prostate cancer properties have also been assessed^20^. The phenolic components have been identified as one of the main *S. crispus* bioactive compounds, in addition to the catechins, alkaloids, caffeine, and tannins, which further contribute to the total defense mechanism. Wasman and Mahmood reported the protective effect of *S. crispus* on gastric ulcers^21^,^22^, but the potential effect of *S. crispus* for preventing colorectal cancer formation has not been studied.

## Results

### *S. crispus* exhibited DPPH free radical scavenging activity

The ability of *S. crispus* to reduce the free radical scavenging activity was tested by assaying DPPH. The DPPH absorption is attributed to the donation of an electron or hydrogen to a stable free radical that is observed *in vitro* as fading of the purple coloration of the absorption band. Our results demonstrated a significant difference between the obtained values of *S. crispus-*treated samples (approximately 86% at 25 μg/ml) and control samples. *S. crispus* exhibited dose-dependent free radical scavenging activity (Figure S1). *S. crispus* showed an IC_50_ value of 5.44 ± 1.76 μmol/l, which was closer to that of vitamin C (3.882 ± 0.628 μmol/l) (Figure S1, Table 1). *S. crispus* ethanol extract eradicated the overproduction of oxygen free radicals and significantly recovered the DPPH free radical scavenging activity.

### *S. crispus* reduced ferric oxidation and decreased the level of nitric oxide radicals

Nitric oxide and ferric acids are very unstable radical species that produce stable products i.e. nitrate, nitrite, ferrous when reacting with oxygen molecules. The oxidation effect of *S. crispus* was tested *in vitro* under specific emission and excitation range and relative to the reported IC_50_ value. Figures S2,S3, and Table 1 illustrate the variation of the scavenging activity with maximum effect in samples treated with *S. crispus* compared to gallic acid (GA), whereas vitamin C exhibited less effect at the same concentration. The ethanol extract of *S. crispus* exhibited a scavenging activity against nitric oxide radicals that expressed an IC_50_ of 0.39 ± 0.11 μmol/l compared to vitamin C and GA (IC_50_ of 0.529 ± 0.009 and 2.78 ± 0.13 μmol/l, respectively). Supplementation with the ethanolic extract of *S. crispus* decreased the reactive oxygen species profiles by decreasing the nitric oxide parameters.

A two-fold higher effect on the reduction of iron from the ferric (Fe^3+^) to ferrous (Fe^2+^) form was observed with *S. crispus*-treated samples relative to BHT and vitamin C (12085.7 ± 0.006, 5228.6 ± 0.01, and 1181.4 ± 0.006 mmol/g, respectively) (Figure S2, Table 1). The cytotoxic effect of reactive oxygen species and the intercellular liberation of nitric oxide directly or indirectly affected the balance between the ferric (Fe^3+^) and ferrous (Fe^2+^) derivatives. Thus, in the *S. crispus-*treated model, the reduction of the ferric (Fe^3+^) to ferrous (Fe^2+^) form was remarkably increased compared to that observed with other conventional antioxidant agents.

### Phenol and flavonoid contents were identified from the plant ethanolic extract

*S. crispus* ethanol extract was evaluated for its total phenolic and total flavonoid content. The TPC was 737.67 ± 0.024 mg (Figure S4-a) and was expressed as gallic acid equivalent in mg per g of plant ethanol extract. The TFC was 262.86 ± 0.005 mg (Figure S4-b) and was expressed as quercetin equivalent in mg per g of plant ethanol extract (Table 1).

### Sublethal dose of *S. crispus* showed no toxicity when orally administered

*S. crispus* at a dose of 2500 mg/kg was nontoxic. No mortality or visible manifestations of hepatotoxic and nephrotoxic effects were observed within the 14 days of observations. Furthermore, there were no significant differences in blood parameters (urea, creatinine, total protein, albumin, glucose and enzymes (ALT, AST, ALP)) between the control and treated groups. Oral administration to rats was therefore considered safe. This gives a wide safety margin for the dosage of *S. crispus* extracts in the tested animals as there were no notable side effects associated to higher doses.

### *S. crispus* supplementation affected body and colon weights

The initial body weights of rats were measured at the start of the experiment and thereafter weekly, spanning the two weeks of induction and eight weeks of treatment. Apart from the weight gain in the animal group treated with 250 mg/kg *S. crispus*, the average weights of 500 mg/kg *S. crispus*-treated and 5-FU drug-treated rats were similar to those of the AOM induced group (Table 2). The colon weights of *S. crispus-*treated groups (both doses) were significantly increased compared to the AOM induced group. In general, there were no significant differences in the liver, spleen and kidney weights among all groups. *S. crispus* induced an increase in the colon and body weight, which might have enhanced the stability of the intestinal wall and therefore improved the removal of superoxide radicals. The integrity of the lumen displayed catalase decomposition of hydrogen peroxide to water and oxygen; thus, allowing the intestinal enzymes to modulate the redox state of lysate and plasma.

### *S. crispus* ethanol extract induced a decrease in ACF number in rat colon

**Figure 1:**
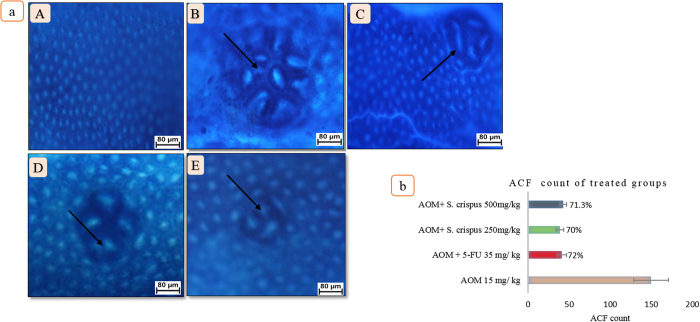
(**a**) Effect of *S. crispus* on macroscopically apparence of ACF in rats’ colon induced by AOM: (A) Normal group with normal crypts, (B) AOM group (ACF with multiple crypts >five), (C) 5-FU group, (D) *S. crispus* 250 mg/kg plant treated group (ACF with crypts <five), (E) *S. crispus* 500 mg/kg plant treated group (ACF with crypts <five) (methylene blue staining) The arrows indicated the crypts that were more than five foci in AOM control group and less than five foci in all treated groups. (**b**) Effect of *S. crispus* on AOM-induced ACF number in rat colon.

Aberrant crypt foci (ACF) were visualized as slightly elevated connective tissue above the surrounding mucosa and demonstrated characteristic oval or slit-like orifices. The intestinal lumen had irregular luminal, elongated protruded crypts. The ACF was observed predominantly in the middle and distal colon in AOM-treated animals, and to a lesser extent in the proximal colon (Fig. 1). AOM induction in the animal models leads to mitochondrial dysfunction and inhibits the release of protective intestinal acids. This was accompanied by the increase of the reactive oxygen species content in the intestinal lumen, contributing to hypertrophy of the cells and deformity of the glands followed by the ACF formation.

All rat groups treated with AOM developed ACF. Table 3 summarizes the effect of *S. crispus* on AOM-induced ACF formation. *S. crispus* produced a marked decrease in the number of ACF (approximately 71–74% of the AOM-induced values) (P < 0.01). *S. crispus* supplementation attenuated the distribution of ACF throughout the colon segments. ACF was mainly found in the middle and distal parts of the colon in the ethanol extract-treated groups and in the middle and proximal areas of the colon in the AOM-induced and 5-FU-treated groups. *S. crispus* treatment resulted in restoring the low number of crypts per focus compared to the AOM-induced rats, as shown in (Table 4). The decrease in the number and size of ACF in *S. crispus-*treated animals indicated that the extract restored the anatomical integrity and decreased the free radical generation, which may be due to the increased antioxidant defense. The average number of ACF in total and per foci decreased remarkably in the group of animals treated with *S. crispus.* The decline in the crypt formation was dominant in animals given *S. crispus* compared to positive and negative control. The above findings may constitute another evidence on how *S. crispus* addition contributes towards the cellular defense mechanisms against free radicals.

### *S. crispus*-treated rats recovered normal intestinal anatomy and histopathological features

**Figure 2:**
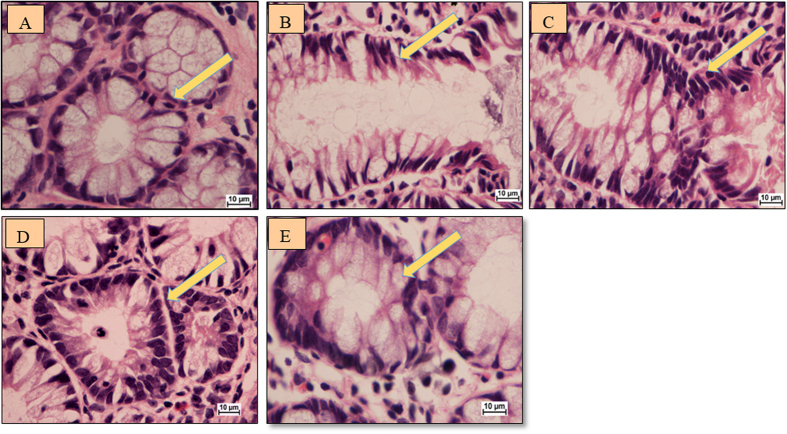
Cross-section of the rat colon stained with hematoxylin and eosin. (**A**) Normal group with normal crypts, (**B**) AOM group (**C**) 5-FU group, (**D**) *S. crispus* 250 mg/kg plant treated group, (**E**) *S. crispus* 500 mg/kg plant treated group (100× magnification). Arrow is indicating to the normal colon cells with round nuclei in normal and treated groups while it is indicating the ACF cell with elongated nuclei and depletion of mucus in AOM induced group.

The histology of ACF and normal colon cells was observed using H&E staining. The colon sections showed normal architecture with intact mucosal and submucosal features. Proliferating mucosal glands and the presence of stained cells with elongated stratified nuclei and depletion of mucus were observed in the colon sections of AOM-induced rats (Fig. 2). It may be relevant that the increase in colon weight was consistent with the reaction of *S. crispus* towards the free radicals, suggesting that *S. crispus* played a role in intestinal homeostasis. The long term oral administration of *S. crispus* revealed no histopathological changes of liver and kidney in plant treated groups with respect to the normal (vehicle) group (Figure S5).

### *S. crispus* ethanol extract decreased the level of MDA and NO, and increased the GPx and CAT activities

Radical species play a crucial role in oxidative stress and their excessive generation is believed to react at the mitochondrial system and produce up- and down-regulation of key proteins that have toxic effects in the biological tissues. We tested the indirect suppressive effect of *S. crispus* on the radical species and the secondary expression of critical cellular proteins. For example, MDA is an aldehydic product of lipid peroxidation that reacts quickly with biomolecules, such as proteins, lipids and nucleic acids and leads to cellular dysfunction. On the other hand, the enhanced interaction of superoxide with NO in the oxidant environment increases the formation of peroxynitrite, which in turn oxidizes tetrahydrobiopterin (BH4, a cofactor for eNOS), leading to eNOS uncoupling. The uncoupling of eNOS, increase in nitric oxide synthase (iNOS) expression and diminished NO bioavailability play major roles in the onset of colon cancer formation. *S. crispus* and 5-FU caused a remarkable decrease in the level of MDA and lipid peroxidation. The group treated with *S. crispus* supplementation was found to normalize the formation of oxygen-derived free radicals. The levels of nitric oxide were significantly decreased in *S. crispus*-treated groups compared with the AOM induced group and the 5-FU reference group. Elevation of GPx and CAT activities was observed in both the AOM induced group and reference 5-FU treated group more than the *S. crispus*-treated groups (Table 5). The decline in MDA and NO activities accompanied by a significant increase in GPx and CAT and the overall change of the antioxidant markers indicate a positive effect of *S. crispus* against the oxidative stress that may also occur in all rat tissues.

### *S. crispus* ethanol extract resulted in faint methylene blue staining of ACF

Aberrant crypt foci (ACF) were visualized as slightly elevated connective tissue above the surrounding mucosa and demonstrated characteristic oval or slit-like orifices. The intestinal lumen had irregular luminal, elongated protruded crypts. The ACF was observed predominantly in the middle and distal colon in AOM-treated animals and to a lesser extent in the proximal colon (Fig. 1). AOM induction in the animal models leads to mitochondrial dysfunction and inhibits the release of protective intestinal acids. The increase in the reactive oxygen species content in the intestinal lumen contributes to cell hypertrophy and deformity of the glands followed by ACF formation. The above findings may constitute additional evidence of how *S. crispus* participates in cellular defense mechanisms against free radicals.

### Low expression of PCNA and Bcl2 staining in the *S. crispus*-treated groups

**Figure 3:**
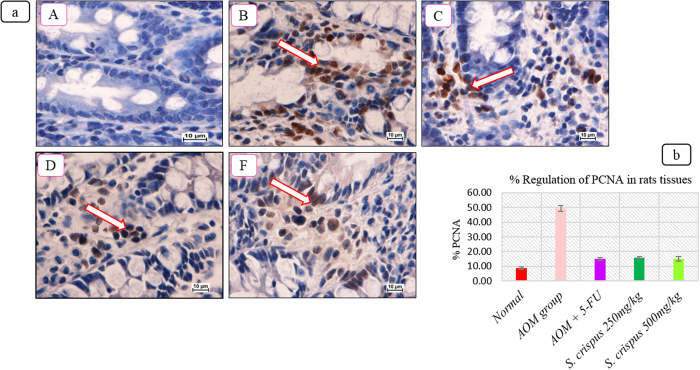
(**a**) Effect of *S. crispus* on regul**a**tion of PCNA protein in the colon tissue of rats (A) Normal group with normal crypts, (B) AOM group, (C) 5-FU group, (D) *S. crispus* 250 mg/kg plant treated group, (E) *S. crispus* 500 mg/kg plant treated group (100× magnification). Arrow indicates cells with PCN protein. (Immunohistochemical stain, PCNA stain). (**b**) Effect of *S. crispus* on % regulation of PCNA protein in AOM-induced rats’ colon tissue.

**Figure 4:**
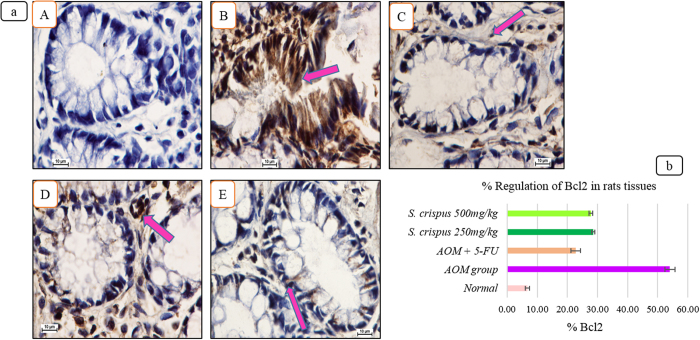
(**a**) Effect of *S. crispus* on regul**a**tion of Bcl2 protein in the colon tissue of rats (A) Normal group with normal crypts, (B) AOM group, (C) 5-FU group, (D) *S. crispus* 250 mg/kg plant treated group, (E) *S. crispus* 500 mg/kg plant treated group (100× magnification). Arrow indicate cells with Bcl2 protein. (Immunohistochemical stain, Bcl2 stain). (**b**) Effect of *S. crispus* on % regulation of Bcl2 protein in AOM-induced rats’ colon tissue.

Representative photographs of the immunohistochemical staining of PCNA and Bc12 positive cells in AOM-treated groups alone, 5 FU-induced AOM-treated groups and *S. crispus-*induced AOM-treated groups clearly show that *S. crispus* suppress the proteins expression. Immunohistochemical results showed that the AOM-induced group had a higher expression of PCNA and Bcl2 protein, seen as heavy brownish staining (Figs 3 and 4), compared to the 5 FU and *S. crispus*-treated groups. The obvious bluish appearance indicated down-regulation of the PCNA protein. The supplementation with *S. crispus* modulated antioxidant gene expression and down-regulated intracellular genes, leading to lower mitochondrial oxygen radical generation.

### Suppression of beta catenin staining in the *S. crispus*-treated groups

**Figure 5:**
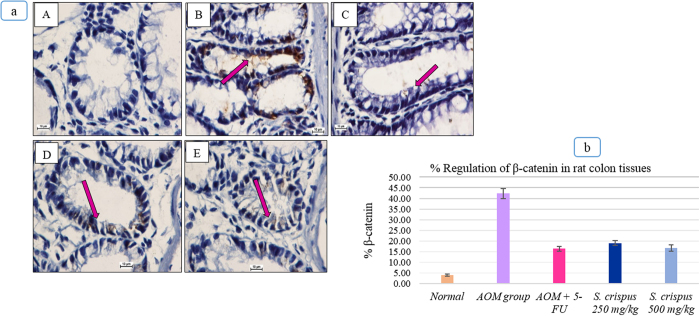
Effect of *S. crispus* on the regulation of β-catenin protein in the colon tissue (A) Normal group (B) AOM control group (C) FU treated group, (D) 250 mg/kg *S. crispus* treated group, and (E) 500 mg/kg *S. crispus* treated group (100× magnification). In the AOM and treated groups the arrows indicated the cells expressed the β-catenin protein. The β-catenin showed to be more expressed in AOM control group than that of treated groups. (Immunohistochemical stain, Bcl2 stain). (**b**) Effect of *S. crispus* on % regulation of β-catenin in AOM-induced rats’ colon tissue.

Beta catenin is a multifunctional protein complex that is a component of cell-to-cell adhesion and part of the WNT signaling pathway. Beta catenin is identified also as one protein of DNA polymerase and its high expression suggests either cellular proliferation (turn over) or DNA mutation. Accumulation of beta-catenin in the nucleus was detected in almost all the AOM-treated groups (Fig. 5). Beta-catenin is a key factor in colorectal carcinogenesis and it is mostly expressed in the membrane at an early stage of the cancer, while the nucleus translocation occurs at an advanced stage illustrating the stages of genetic mutations. The labeling index of beta catenin in the colonic mucosa is presented in Fig. 5. Qualitative analysis of the stained sections following AOM- treatment showed a clear elevation and translocation of the protein to the colonic cancer cells compared with groups treated with 5 FU and *S. crispus*. Microscopic examination of the colonic tissue sections stained for beta catenin clearly showed a faint or no staining in *S. crispus-*induced AOM-treated groups.

## Discussion

We investigated the chemopreventive effect of *S. crispus* ethanol extracts on AOM-induced colorectal lesions by *in vivo* and the scavenging activity for free radicals by *in vitro* assays. AOM has been commonly used as a carcinogenic method to determine the chemopreventive effects of food ingredients such as indigestible sugars, red meat, and green tea in rodent models^23^. We used the AOM experimental approach^24^ to initiate colon cell outgrowth that mimics the cryptal foci lesions in cancers. The AOM carcinogenic effect is largely reported in the literature by other groups of researchers^25^, as well as in our work^9^. In fact, we had already validated this approach from histopathological and biochemical perspectives in a previous study^26^. The dose safety and efficacy had been evaluated previously^18^ and in the present study and in accordance with the highest recommended dose, and the animals were healthy and showed no hepatotoxicity and/or nephrotoxicity. Moreover, because an objective of the present work was to study the pharmacological effects of *S. crispus,* a simple acute model was useful.

*S. crispus* ethanol extract reduced the expected aberrant cryptal foci histological deficit, restoring the anatomical appearance of the wall as well as the physiological performance of mucus production. Histologically, the AOM-induced lesions showed proliferating mucosal glands with ACF characterized by elongated stratified nuclei, depletion of mucin and damage of submucosal glands, which is characteristic of benign aberrant focal crypti. In contrast, our observations showed a decrease in the number and size of aberrant cryptal foci with the *S. crispus* treatment. Immunohistochemical staining showed increased binding activity of PCNA and Bcl2 proteins and nuclear translocation with a concomitant deformity of the mucosal and submucosal features. Enhancement of PCNA and Bcl2 expression after AOM application, but not with *S. crispus* administration, was correlated with the proliferation process and the variation of mucosal thickness. PCNA and Bcl2 play an important role in DNA synthesis and DNA repair. PCNA and Bcl2 antibodies are commonly used for grading neoplasms^27^,^28^. It is well known that AOM produces loss of mucosal integrity and decreases the power of releasing mucin, accompanied by deficits in the submucosal glands^29^. Introduction of *S. crispus* ethanol extracts before AOM seems beneficial in retarding colorectal cancer formation by preventing cell proliferation and inhibiting the activation of PCNA and Bcl2. In conclusion, the integrity of the intraluminal wall structure is fundamental from both a protection and absorption prospective, as it contributes to the encoding of physiological and anatomical balance, hinders the transmission of harmful chemical signals and is essential for maintaining the flow in the mucous layer on the intraluminal surface. Therefore, it is not surprising that *S. crispus* supplementation increased the colon and total animal weights compared to other treatment groups.

Colorectal cancer, among other serious cancers, is correlated with oxidative insults. *In vitro* evidence shows that AOM administration generates a toxic cascade that mediates the apoptotic pathway in culture models^30^. Our data indicated the scavenging effect of *S. crispus* in the treated samples. *S. crispus* inhibits oxidative stress via attenuation of apoptotic signaling pathways in the cell. The high potent activities of *S. crispus* were due to the deactivation of the metabolic redox correlated directly with its suppressing effect on mitochondrial and nuclear levels. The *S. crispus* ethanol extract was shown to attenuate the toxic action of oxidative free radicals induced by the demonstration of AOM. The effect was higher than that of the anti-oxidative agents (i.e., vitamin C, gallic acid, quercetin and butylated hydroxytoluen) as assessed by DPPH, ferric reducing and nitric oxide scavenging assays. AOM supplementation induced the accumulation of free radicals and increased the intracellular level of apoptotic-sensitive proteins. The free radical scavenging mechanism involves oxidative-mediated apoptosis, which requires ferric acid reduction; therefore, the production of NO and iNOS is regulated^31^. *S. crispus* at an IC50 of 5.44 + 1.76 effectively inhibited DPPH free radicals, nitric oxide and ferric reduction sequences. These findings are supported by other studies that have shown decreased MDA^31^ and NO levels^32^ in rats treated with *Strobilanthes asperrimus*. Moreover, the measurements of serum biomarkers following oxidative stress showed that the levels were within the normal range and suggested a possible role of *S. crispus* to normalize the level of serum biomarkers (i.e., urea, creatinine, total protein, albumin, glucose and enzymes (ALT, AST, and ALP)). The apoptotic signaling pathway of AOM induced a deleterious increase in the apoptosis-sensitive proteins (specifically MDA, GPx and CAT, among others) in the tissue, which were attenuated by *S. crispus*.

The intestinal microenvironment after chemical erosions by AOM seems to produce the second messenger nitric oxide (NO) following the excessive release of inducible nitric oxide synthase (iNOS). The production of iNOS is Ca^2+^-independent and known to participate in and inhibit the initiation of certain diseases. This process altered specific genes expression as well as a wide range of proteins and signaling cascades^31^,^32^. For instance, ROS release causes lipid peroxidation of polyunsaturated fatty acids and damage to specific mitochondrial proteins and transport systems by direct inhibition of enzymes resulting in loss of mitochondrial integrity. Malondialdehyde (MDA), one of the final products of polyunsaturated fatty acids peroxidation, is commonly known as a marker of oxidative stress and reflects the antioxidant status. In the present study, the data showed that MDA levels significantly decreased, indicating that *S. crispus* stopped the lipid peroxidation that is the prominent player in the membranes damage. Previous studies have demonstrated that the primary antioxidant enzymes which include SOD, GPx and CAT generate the antioxidant defense systems. The antioxidant enzymes provide the first line of cellular defense against ROS. In the present study, the data showed that SOD, GPx, and CAT significantly increased leading to reduction of the free radical damage. Additionally, PCNA and Bc12 genes are produced by enzymatic cleavage and considered as a biomarker of oxidative DNA damage. Increased levels of DNA damage can cause the synthesis of a variety of incorrect proteins and therefore mutations and cancer formation.

At least two models for genetic alterations can be characterized in the development of colorectal cancer. In the presence of β-catenin staining, the scenario can be that the transcriptional effects were initiated by Rac1-dependent nuclear translocation of β-catenin followed by mutation of the Wnt ligand, and deleted in colorectal cancer (DCC). The WNT signal transduction can also transcriptionally activate both NOS2 and COX2 by TCF4-β-catenin binding to their promoters in a regulatory feedback loop. Under these conditions and secondary to the release of iNOS, K1 or 2 kinases and GSK3beta phosphorylated β-catenin and activated threonine and serine residues in the N-terminal domain. Conversely, the NO-related DNA damage leads to p53 accumulation and p53-mediated apoptosis. NO can activate the WNT pathway by enhancing the enzymatic activity of cyclo-oxygenase 2 (COX2 and the release of prostaglandins 2E (PGE2))^25^. Beside these two pathways there is a possibility that less characterized co-activators/co-repressors displayed the transcription with a high degree of overlap among the main pathways.

Finally, recent studies have opened a new perspective for the possible use of *S. crispus* in medicine^18^,^22^. In fact, experimental data clearly indicated a significant protective role of *S. crispus* in breast cancer^20^. These findings are consistent with previous studies reporting the phenolic content and antioxidant activity of this plant^15^,^16^,^33^. Moreover, a recent study demonstrated the phenolic content and cytotoxic potential role of *S. crispus*^16^,^34^. Accordingly and consistent with this research, our laboratory recently reported, *in vivo*, using rats, a significant inhibition of colorectal cancer formation after similar treatment with medicinal plants (*Andrographis paniculata* and *Gynura procumbens*)^9^,^25^. Our results demonstrated the chemoprotective efficiency of *S. crispus* ethanol extracts, which can be attributed to its anti-proliferative, antioxidant and anti-radical scavenging properties. Accordingly, this natural agent might provide a potential template for the design of drug targets for treating colorectal cancers.

## Materials and Method

### Chemicals

Azoxymethane (Sigma Chemical Co., St Louis, MO, USA) was dissolved in sterile 0.9% normal saline and was given subcutaneously at 15 mg/kg body weight once a week for 2 weeks to induce ACF^35^. Fluorouracil (5-FU) (Sigma Chemical Co., St. Louis, MO, USA) was dissolved in 0.9% normal saline and was administered at a concentration of 35 mg/kg body weight intraperitoneally twice a week^36^.

### Plant materials

The fresh plant leaves were obtained from Ethno Resources Sdn. Bhd., Selangor, Malaysiy, and identified by a voucher specimen placed at the Herbarium of Rimba Ilmu, Institute of Biological Sciences, University of Malaya, Kuala Lumpur. The leaves were washed, shade-dried and grounded. The powdered plant material (100 g) was extracted with ethanol 95% (1000 ml) at room temperature three times, three days per each crop with occasional stirring and filtered. Three extracts were combined and evaporated in vacuo to give 11.5 g of crude ethanol extract^37^ which was kept dry at 45 °C, and the clear semisolid ethanol extract was then dissolved in the vehicle solution.

### *In vitro* study of the antioxidant properties of *S. crispus* ethanol extract

#### The 2, 2-diphenyl-1-picryl-hydrazyl free radical scavenging assay

The free radical scavenging activity of the plant ethanol extract was determined spectrophotometrically using 2, 2-diphenyl-1-picryl-hydrazyl (DPPH, Sigma-Aldrich, UK). DPPH, which is violet, produces a yellow complex when it is reduced in the presence of antioxidants^38^. The DPPH working solution was prepared using 95% ethanol at a concentration of 3.94 mg/100 ml, and ascorbic acid was used as the standard. The sample and DPPH reagent were mixed well and incubated before the absorbance was measured using an ELISA plate reader at 517 nm in triplicate^39^. The scavenging activity of the ethanol extract was calculated based on the percentage of scavenged DPPH. (BIO-TEK instruments, Winooski, VT, USA).

#### Ferric-reducing antioxidant potential (FRAP) assay

The ferric-reducing antioxidant potential (FRAP, Thermo Fisher Scientific, USA) assay was performed as previously described^40^. FRAP reagent was prepared by mixing 16.7 mM FeCl_3_.6H_2_O and 8.3 mM 2,4,6-tripyridyl-s-triazine (TPTZ) with 250 mM acetate buffer, pH 3.6. The ethanol extract absorbance was read at zero minutes and at 4 minutes at 593 nm relative to FeSO_4_.7H_2_O as the standard negative control at 400–1000 mM by using a microplate reader. Vitamin C, gallic acid (GA), butylated hydroxytoluene, and quercetin were used as positive controls^39^.

#### Nitric oxide scavenging activity

Nitrite (NO_2,_) is the stable product of nitric oxide conversion as assessed using Griess reagent (Sigma-Aldrich, UK). A series of dilutions were prepared from a 1 mg/ml stock of *S. crispus* ethanol extract. For every concentration, 50 μl of the ethanol extract was transferred into a 96-well microplate, and an equal volume of sodium nitroprusside was added prior to the addition of Griess reagent (100 μl). After 5 minutes, the absorbance was read using a microplate reader at 550 nm under visible polychromatic light^41^ using sodium nitrite as the standard. The percentage of inhibition was obtained by calculating the ability of the plant ethanol extracts to inhibit nitric oxide formation relative to 0% inhibition of the control^42^. This ability was defined as (Ao − As/Ao)*100, where Ao is the absorption of control, and As is the absorption of the test samples.

#### Total phenolic and total flavonoid content of the plant ethanol extract

Total phenolic content (TPC) was determined according to the Folin-Ciocalteu (Thermo Fisher Scientific, USA) spectrophotometric method with slight modifications using a microplate reader. The measurement was compared to a standard curve of GA solution, and the value was expressed as milligrams of GA equivalent (GAE) per gram of dry plant ethanol extract (mg GAE/g db)^43^. The total flavonoid (TFC) content was measured using the aluminum chloride colorimetric method. The measurement was expressed as a quercetin equivalent in mg (QE)/g of ethanol extract^44^. The assays were performed in triplicate.

### *In vivo* study

#### Ethical approval

The animal studies were approved by the Ethical Committee for Animal Experimentation, Faculty of Medicine, University of Malaya, Malaysia; [Ethic No. PM/07/05/2011/MMA (a) (R) for acute toxicity study and PM/07/05/2012/MMA (b) (R) for chemopreventive study]. Throughout the experiments, all animals received care according to the criteria outlined in the “Guide for the Care and Use of Laboratory Animals” (2011).

#### Experimental animals

Healthy adult SD female rats were used for the acute toxicity study, whereas male rats were used for the chemopreventive study. Rats (6–8 weeks old), weighing between 150–180 g, were placed individually in separate cages. The animals were maintained on a standard diet and tap water ad libitum, under controlled conditions of humidity (50–60%), lighting (12-h light/dark cycle) and temperature (22–24 °C), and they were weighed weekly.

#### Acute toxicity study

A total of 16 healthy SD female rats were obtained from the Experimental Animal House, Faculty of Medicine, University of Malaya. The animals were divided equally into two groups: a) treated with vehicle (10% Tween-20, Thermo Fisher Scientific, USA) and treated with 2500 mg/kg ethanol extract in vehicle, respectively. The animals were fasted overnight (food but not water) prior to dosing. Food was withdrawn for a further 3 to 4 hours, and the animals were observed for 30 min and at 2, 4, 24 and 48 h for the onset of clinical or toxicological symptoms. Mortality, if any, was noted over a period of 2 weeks; then, the animals were sacrificed on the 15th day. The colon, liver and spleen weights were reported. Serum biochemical and histological (liver and kidney) parameters were determined following standard methods^45^.

#### Experimental design

Following the safety/toxicity test and within the range of the chosen safe dose, we divided the adult male SD rats randomly into the following five groups:-

Group 1: (Saline group) injected with 0.9% sterile normal saline once a week for two weeks and fed daily with 10% Tween-20 (Thermo Fisher Scientific, USA) (5 ml/kg) for 60 days.

Group 2: (AOM group) injected with (15 mg/kg/ml) of AOM once a week for two weeks and fed daily with 10% Tween-20 (5 ml/kg) for 60 days.

Group 3: (5-FU group) injected with (15 mg/kg/ml) of AOM once a week for two weeks and injected intraperitoneally with (35 mg/kg/ml) of 5-FU twice weekly eight weeks.

Groups 4 & 5: (Plant treated groups) injected with (15 mg/kg/ml) of AOM once a week for two weeks and fed daily with 250 mg/kg and 500 mg/kg (5 ml/kg) of ethanol extract for 60 days^36^,^46^.

#### Histopathological examination

All rats were sacrificed by cervical dislocation after receiving an overdose of ketamine and xylazine (50 and 5 mg/kg body weight, respectively). Colons were removed and washed with cold phosphate buffered saline (PBS, Thermo Fisher Scientific, USA). They were then dissected longitudinally and cut into three equal segments (proximal, middle, and distal). The segments were fixed flat between filter paper in 10% formalin for 5–10 minutes and stained with methylene blue (Sigma-Aldrich, UK) (0.2% in PBS solution) for 15–20 minutes to visualize the internal surface of the lumen furnished with the crypts. Each segment was further sliced into 2 cm sections and placed on microscope slides with the mucosa facing upwards. The aberrant crypts were recognized by their abnormal sizes and scored under the light microscope at 4× magnification. The number of ACF per colon, the number of aberrant crypts (ACs) in each optical focus, and the location of each focus were recorded^46^,^47^. The paraffin blocks were processed (Leica Microsystem, Nussloch, Germany) and sectioned at 4 μm thickness using a microtome and stained with hematoxylin-eosin (H&E). Sections were examined and imaged with a Nikon camera connected to an Olympus light microscope (Tokyo, Japan).

#### Biochemical markers

Blood samples were collected in gel activating tubes and centrifuged at 3000 rpm for 10 minutes. The serum was separated and sent for determination of creatinine, total protein, glucose, albumin, alkaline phosphatase (ALP), alanine aminotransferase (ALT), aspartate aminotransferase (AST) and urea levels. Biochemical parameters were determined using the standard automated analyzer at the Central Diagnostic Laboratory, University of Malaya Medical Center.

#### Determination of antioxidants in colon homogenate

The colon tissue was washed with ice-cold PBS solution, pH 7.4, to remove other cells or debris. The colon tissue was homogenized on ice and centrifuged at 4500 rpm for 15 minutes at 4 °C. Tissue homogenate supernatant was used to measure the antioxidants^48^,^49^. Protease, a mixture of protease inhibitors with broad specificity for the inhibition of serine, cysteine, aspartic proteases and amino peptidases, was used to preserve enzymes from being degraded. Malonaldehyde (MDA) (cat #10009055) and nitric oxide (NO) (cat #D2NO-100) levels were measured. Glutathione peroxidase (GPx) (cat #703102) and catalase (CAT) (cat #707002) activities were also measured. All measurements were performed using commercial kits (Cayman Chemical Company, U.S.A) on a microplate reader.

#### Immunohistochemical staining

Colon sections were mounted on poly-lysine coated slides, dried overnight in an oven at 60 °C and then deparaffinized and rehydrated in graded alcohols. For retrieval of the antigen target, a retrieval solution containing 10 mM sodium citrate buffer was used. Immunohistochemical staining was done according to the manufacturer’s protocol (Dako ARK™ USA), based on avidin, biotin and peroxidase methodologies. Biotinylated primary antibody of diluted mouse PCNA and Bcl2 monoclonal antibody were applied at 1:100 and 1:50 dilution, respectively, followed by applying streptavidin-peroxidase (streptavidin conjugated to horseradish peroxidase in PBS containing the anti-microbial agent). Proliferating cell nuclear antigen (PCNA) is a well-known indicator of proliferation, and Bcl2 is a well-known anti-apoptotic protein indicator. Staining was completed by a 5-minute incubation with 3, 3’-diaminobenzidine (DAB)-substrate-chromogen. Negative control sections were processed similarly but with the omission of the primary antibodies. Slides were rinsed in a bath of distilled or deionized water for 2–5 min, mounted and cover-slipped with an aqueous-based mounting medium or a non-aqueous permanent mounting.

#### Beta catenin immunostaining

Briefly, sections were deparaffinized and rehydrated through graded alcohol. Endogenous peroxidase activity was blocked with 0.5% hydrogen peroxide in methanol for 20 minutes. Antigen retrieval was used by microwave treatment; dilution was 1:100 for beta catenin (Novocastra, United Kingdom). Biotinylated universal antiserum (Starr Trek HRP Universal, Biocare Medical, CA) was used as the secondary antibody. After washing, the slides were incubated for 20 minutes at room temperature with Trekavidin HRP label (Starr Trek HRP Universal Biomedical CA) and developed for 10 minutes using 3- 3- diaminobenzidine as chromogen. After rinsing in water, the sections were counterstained with Meyer haematoxylin, dehydrated, and mounted. Appropriate positive and negative controls were included in each run of immunohistochemistry.

### Statistical analysis

Values were expressed as the mean ± SEM. Variation between groups was investigated employing one-way ANOVA followed by Tukey’s post-hoc test using SPSS version 20 (SPSS Inc. Chicago, IL, USA). P values less than 0.05 were statistically significant when compared to the control group.

## Additional Information

**How to cite this article**: Al-Henhena, N. *et al.* Chemopreventive effects of *Strobilanthes crispus* leaf extract on azoxymethane-induced aberrant crypt foci in rat colon. *Sci. Rep.* **5**, 13312; doi: 10.1038/srep13312 (2015).

## Supplementary Material

Supplementary Information

## Figures and Tables

**Table 1:** Antioxidant activity and total flavonoids and phenolic of *S. crispus* crude extract *in vitro*.

	BHT	Vitamin C(×10)	Gallic acid	Quercetin	*S. crispus*
DPPH (IC_50_ μg/ml)*	10.12 ± 0.76	3.882 ± 0.628	…….	…….	5.435 ± 0.76
FRAP (nM/g)	5228.6 ± 0.01	1181.4 ± 0.006	…….	…….	12085.7 ± 0.006
TPC (mg/g)	…….	…….	1015.3 ± 0.004	…….	737.7 ± 0.024
TF (mg/g)	…….	…….	…….	775.71 ± 0.009	262.86 ± 0.0009
NO (IC_50_ μg/ml)*		0.529 ± 0.001	2.78 ± 0.013		0.39 ± 0.002

Values were represented as mean ± SEM for triplicates; BHT-butylated hydroxytoluene.

**Table 2:** Effect of *S. crispus extract* on weights of the body, liver, colon, spleen and kidney in AOM induced ACF in rats.

Group	Body	Colon	Liver	Spleen	Kidney
Normal	307.33 ± 12.65	2.62 ± 0.05	8.16 ± 0.23	0.88 ± 0.05	2.51 ± 0.15
AOM (15 mk/kg)	314.50 ± 19.56	3.14 ± 0.24	9.87 ± 0.67	0.86 ± 0.05	2.82 ± 0.36
AOM + 5-FU (35 mg/kg)	383.50 ± 17.38	2.18 ± 0.16	10.09 ± 0.05	0.66 ± 0.03	2.93 ± 0.07
AOM + *S. crispus* (250 mg/kg)	413.33 ± 23.05*	4.62 ± 0.52*	10.77 ± 0.75	0.98 ± 0.06*	2.48 ± 0.32
AOM + *S. crispus* (500 mg/kg)	397.50 ± 31.83	3.95 ± 0.44*	11.17 ± 0.52	1.03 ± 0.12*	2.49 ± 0.30

Values expressed as mean ± S.E.M. *Significant difference at a level of p < 0.05. 5-FU: 5-Fluorouracil; AOM: azoxymethane.

**Table 3:** Effect of *S. crispus* extract on AOM-induced ACF number in rat colon.

Group	ACF		ACF distribution at colon segments		
	Total	Inhibition %	Proximal	Middle	Distal
Normal [10% Tween-20 5 ml/kg]	0	0	0	0	0
AOM 15 mg/kg	150 ± 21.0	0	37 ± 4.60	83 ± 14.5	30 ± 9
AOM + 5-FU 35 mg/kg]	41 ± 9.6**	72.6%	5 ± 1.2**	32 ± 9.74**	4 ± 1.7**
AOM + *S. crispus* (250 mg)	44 ± 4.6**	70.6%	8 ± 0.58**	20 ± 1.1**	16 ± 4.1**
AOM +*S. crispus* (500 mg)	43 ± 2**	71.3%	10 ± 1.76**	19 ± 2.7**	14 ± 3.4**

All values are expressed as mean ± S.E.M. Mean with different superscript are significantly different at (p < 0.05). 5-FU-Fluorouracil, ACF-aberrant crypt foci, AOM-azoxymethane.

**Table 4:** Effects of *S. crispus* extracts on the number of crypts per focus in AOM induced ACF in colons of male rats.

Group	Number of foci containing				
	1 crypt	2 crypts	3 crypts	4 crypts	>5 crypts
Cancer control group (AOM)	26 ± 7.15	36 ± 8.4	31 ± 4.4	25 ± 1.4	32 ± 7.3
AOM + 5-FU	10 ± 2.1**	9 ± 2.3**	9 ± 2.6**	5 ± 1.5**	8 ± 2.6**
AOM + *S. crispus* (250 mg/kg)	4 ± 1.4**	11 ± 2.3**	13 ± 0.5**	8 ± 2.5**	8 ± 2.4**
AOM + *S. crispus* (500 mg/kg)	8 ± 0.5**	10 ± 2.7**	11 ± 2.1**	8 ± 1.2**	6 ± 1.2**

All values are in mean ± S.E.M. **Significant difference at p < 0.01 (ANOVA, Tukey’s post hoc). 5-FU: 5-Fluorouracil; ACF: aberrant crypt foci; AOM: azoxymethane.

**Table 5:** Effect of *S. crispus* on GPx and CAT activities, NO and MDA levels in colon homogenate 60 days after treatment.

Group	Antioxidant activity in homogenate			
	GPX nmol/min/ml	CAT nmol/min/ml	NO μM	MDA μM
Saline group	16.05 ± 1.12	12.88 ± 1.4	26.38 ± 1.12	3.94 ± 0.269
AOM group	13.45 ± 4.76393	11.14 ± 1.06	37.20 ± 6.87	9.98 ± 0.78
AOM + 5-FU	19.29 ± 3.145*	18.91 ± 0.59*	14.92 ± 1.04	3.60 ± 0.12***
AOM + *S. crispus* 250 mg	16.67 ± 1.59*	15.95 ± 0.87*	13.39 ± 2.67**	4.34 ± 0.63***
AOM + *S. crispus* 500 mg	17.43 ± 1.70*	16.04 ± 1.45*	11.64 ± 0.18**	3.21 ± 0.46***

All values are expressed as mean ± SEM. Significant difference at *P < 0.01, **P < 0.001, ***P < 0.0001 (ANOVA, Tukey’s post hoc). 5-FU: 5-Fluorouracil; ACF: aberrant crypt foci; AOM: azoxymethane.
