# Supplementary material for: Chemopreventive effects of Strobilanthes crispus leaf extract on azoxymethane-induced aberrant crypt foci in rat colon
Source: Sci Rep. 2015 Aug 26;5:13312. doi: 10.1038/srep13312 (PMC4642519; doi:10.1038/srep13312)
Supplement: Supplementary Information [file srep13312-s1.pdf]

Chemopreventive effects of *Strobilanthes crispus* leaf extract on azoxymethane-induced aberrant crypt foci in rat colon

Nawal Al-Henhena[1], Shaden A. M. Khalifa[2], Rozaida Poh Yuen Ying[1], Pouya Hassandarvish[1], Elham Rouhollahi[1], Nahla Saeed Al-Wajeeh[1], Habibah Mohd Ali[3], Mahmood Ameen Abdulla[1,*] & Hesham R. El-Seedi[3,2,4,*]

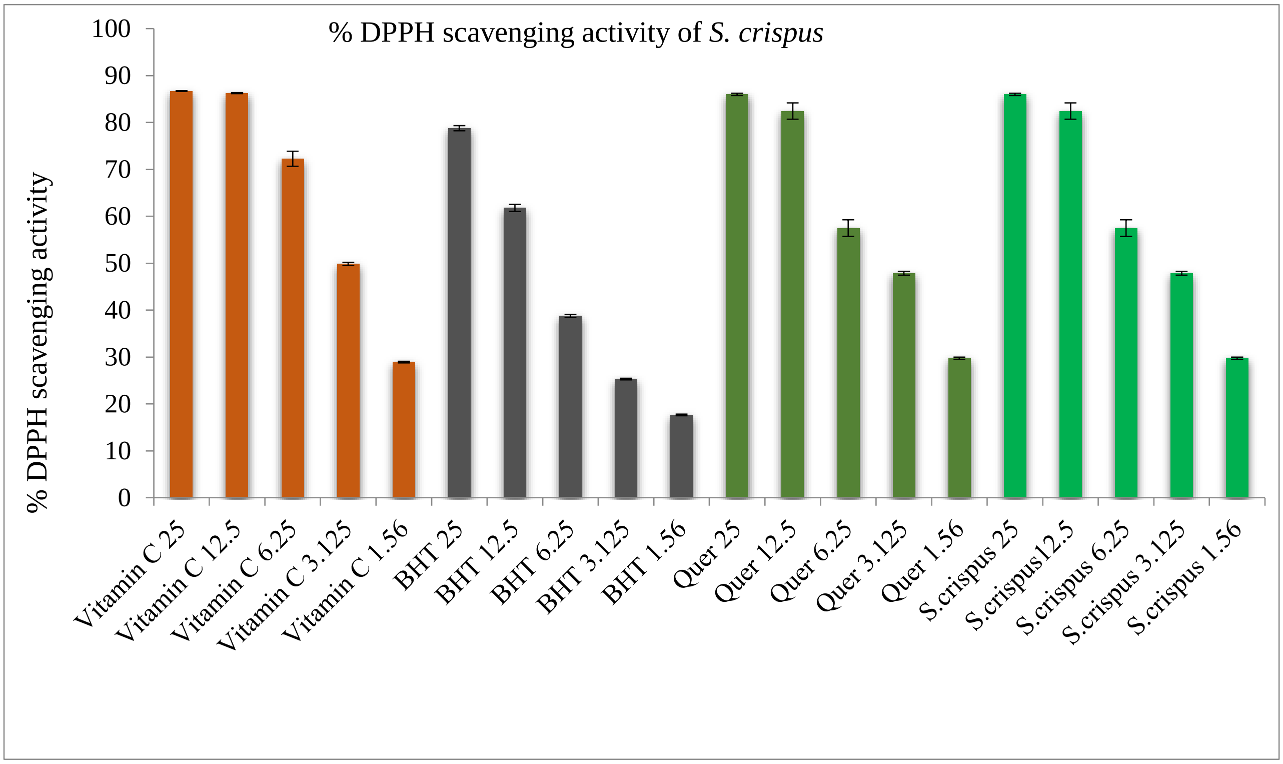

Figure S1: DPPH scavenging activity of crude extracts of *S. crispus* (dilution in µg/ml) in comparison with various known antioxidants. The data are expressed as mean ± SEM for triplicate. BHT-butylated hydroxytoluene, Quer-quercetin.

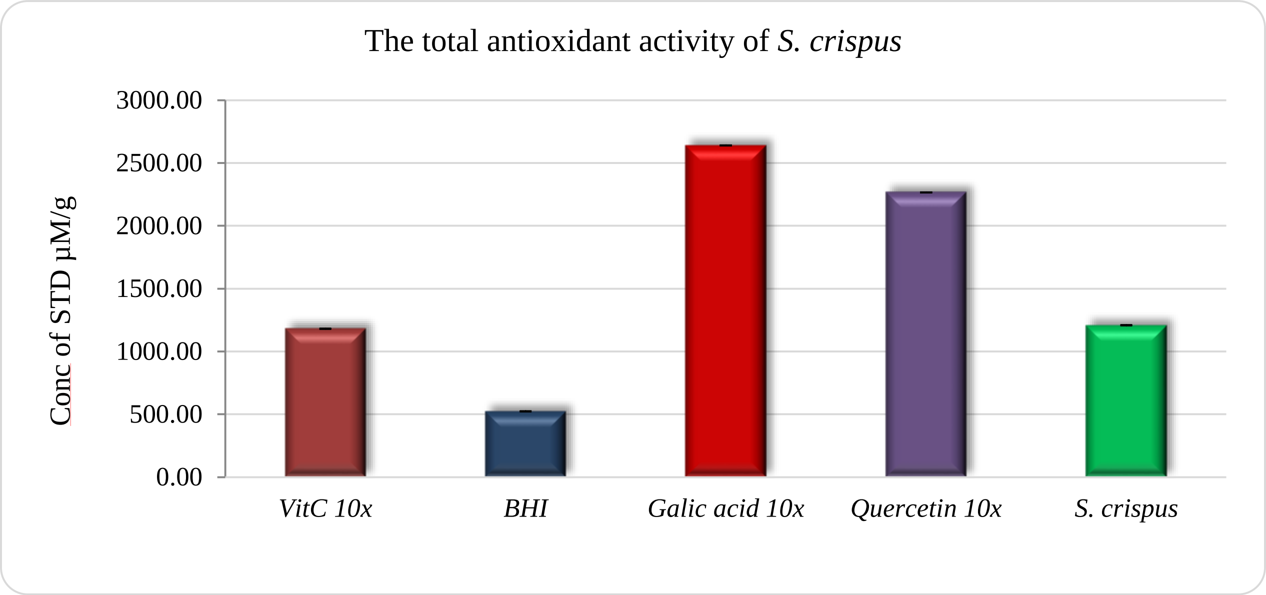

Figure S2: FRAP activity of *S. crispus* extract. The data are expressed as mean ± SEM for triplicate. BHT-butylated hydroxytoluene, VitC: vitamin C.

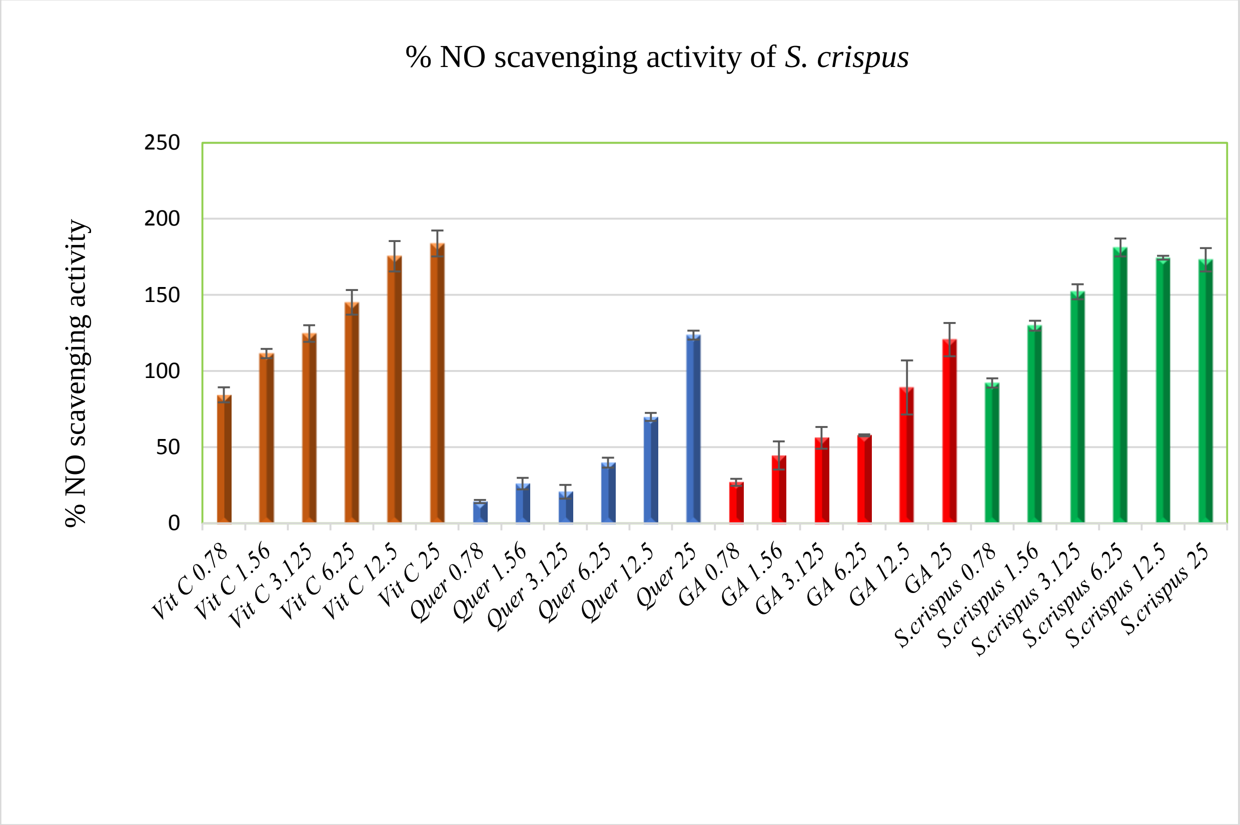

Figure S3: NO Scavenging activity of crude extracts of *S. crispus* (dilution in µg/ml). The data are expressed as mean ± SEM for triplicate. VitC-vitamin C, Quer-quercetin, GA-gallic acid

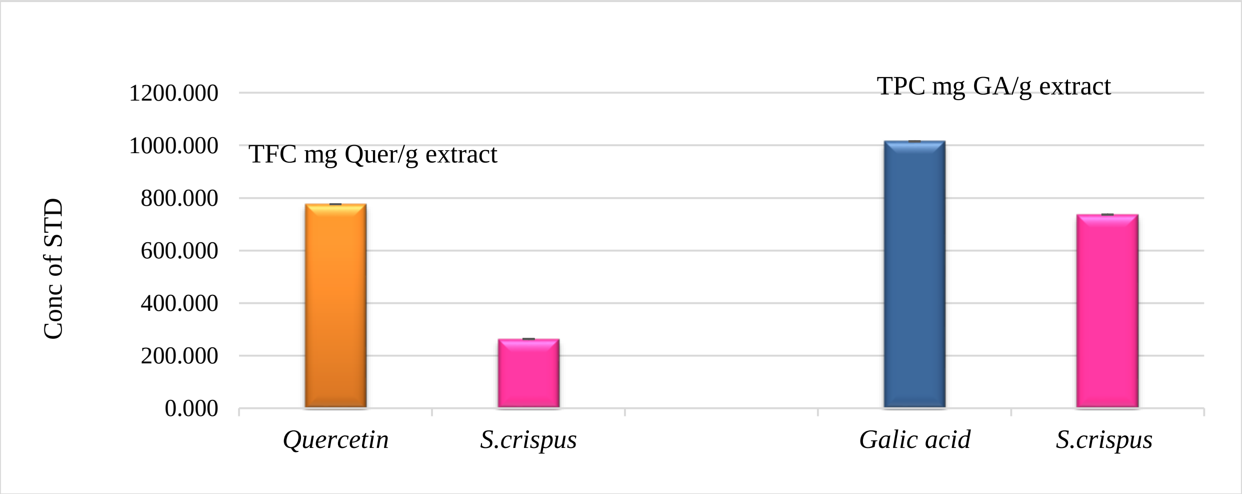

Figure S4: Antioxidant activity of *S. crispus in vitro*. a) Total phenolic content of *S. crispus*, (b) Total flavonoid content of *S. crispus*. Values are represented as mean ± SEM for triplicates

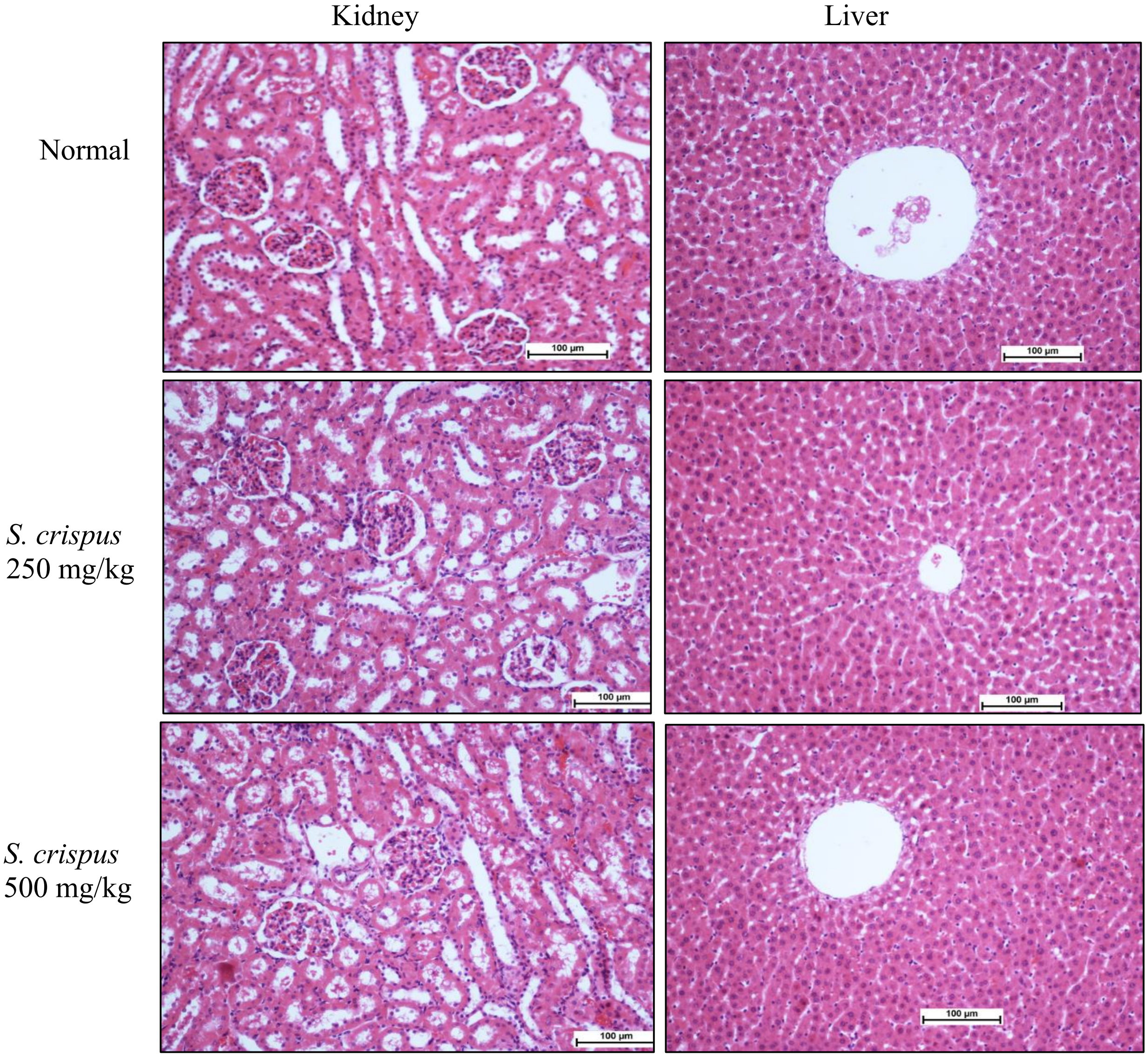

Figure S5. Effect of *S. crispus* on the histological changes of major organs (Kidney and Liver) upon dosing for 60 days. They showed normal and no related toxicity in liver and kidney of *S. crispus*-treated AOM-induced ACF.
